# Research paramedics’ observations regarding the challenges and strategies employed in the implementation of a large-scale out-of-hospital randomised trial

**DOI:** 10.29045/14784726.2020.06.5.1.26

**Published:** 2020-06-01

**Authors:** Jonathan Green, Maria Robinson, Richard Pilbery, Gregory Whitley, Helen Hall, Madeleine Clout, Barnaby Reeves, Kim Kirby, Jonathan Benger

**Affiliations:** University of Plymouth; South Western Ambulance Service NHS Foundation Trust; South Western Ambulance Service NHS Foundation Trust; Yorkshire Ambulance Service NHS Trust; East Midlands Ambulance Service NHS Trust; University of Lincoln; East of England Ambulance Service NHS Trust; James Paget University Hospitals NHS Foundation Trust; Bristol Medical School; Bristol Medical School; South Western Ambulance Service NHS Foundation Trust; University of the West of England; University of the West of England

**Keywords:** emergency medical services, emergency medical technicians, out-of-hospital cardiac arrest

## Abstract

**Introduction:**

AIRWAYS-2 was a cluster randomised controlled trial (RCT) comparing the clinical and cost effectiveness of the i-gel supraglottic airway device with tracheal intubation in the initial airway management of out-of-hospital cardiac arrest (OHCA). In order to successfully conduct this clinical trial, it was necessary for research paramedics to overcome multiple challenges, many of which will be relevant to future emergency medical service (EMS) research. This article aims to describe a number of the challenges that were encountered during the out-of-hospital phase of the AIRWAYS-2 trial and how these were overcome.

**Methods:**

The research paramedics responsible for conducting the pre-hospital phase of the trial were asked to reflect on their experience of facilitating the AIRWAYS-2 trial. Responses were then collated by the lead author. A process of iterative revision and review was undertaken by the research paramedics to produce a consensus of opinion.

**Results:**

The main challenges identified by the trial research paramedics related to the recruitment and training of paramedics, screening of eligible patients and investigation of protocol deviations / reporting errors. Even though a feasibility study was conducted prior to the commencement of AIRWAYS-2, the scale of these challenges was underestimated.

**Conclusion:**

Large-scale pragmatic cluster randomised trials are being successfully undertaken in out-of-hospital care. However, they require intensive engagement with EMS clinicians and local research paramedics, particularly when the intervention is contentious. Feasibility studies are an important part of research but may fail to identify all potential challenges. Therefore, flexibility is required to manage unforeseen difficulties.

## Introduction

AIRWAYS-2 was a cluster randomised controlled trial (RCT) comparing the clinical and cost effectiveness of the i-gel supraglottic airway device with tracheal intubation in the initial airway management of out-of-hospital cardiac arrest (OHCA). It is the largest trial of its kind published to date, and the results have advanced the evidence base for an important area of emergency medical service (EMS) practice (Benger et al., 2018). In order to successfully conduct this clinical trial, it was necessary for the research paramedics (RPs), who were responsible for paramedic recruitment and training, patient screening and data collection, to overcome multiple challenges, many of which will be relevant to future EMS research.

The out-of-hospital environment is a challenging research setting, and EMS clinicians are a mobile workforce operating under significant operational pressures with limited opportunities for training. This may partly explain why fewer than 1% of out-of-hospital studies are RCTs (Venkataraman et al., 2014). However, the trial was led by a chief investigator with experience of ambulance service practice and research, and the ambulance services were involved early in trial set-up. In keeping with good research practice, a feasibility study was conducted (REVIVE-Airways) prior to the main study (Benger et al., 2016), with paramedics involved in the development of the feasibility and main study (Benger et al., 2013; Rhys et al., 2013; Taylor et al., 2016).

The challenges of study set-up, enrolment and follow-up of the AIRWAYS-2 trial have previously been discussed (Robinson et al., 2019). This article aims to describe a number of the challenges that were encountered by the research paramedics during the delivery of the pre-hospital phase of the AIRWAYS-2 trial and how these were overcome.

## Methods

Following completion of the AIRWAYS-2 trial, the four research paramedics (one in each of the participating ambulance services) and the coordinating lead research paramedic were asked to reflect on their experience of facilitating the trial. They were asked to submit their observations, describing challenges encountered when implementing the trial and the strategies employed to overcome them. Responses were provided via e-mail as free-text responses. These responses were then collated by the lead author and underwent a process of iterative revision and review by the research paramedics and other members of the trial management group to produce a consensus of opinion.

## Results

The main challenges identified by the research paramedics responsible for the implementation of the out-of-hospital ‘intervention’ phase of AIRWAYS-2 related to the recruitment and training of paramedics, screening of eligible patients and investigation of protocol deviations / reporting errors.

### Challenges

#### Recruiting and training

In order to enrol the required 9070 patients for the trial, it was estimated that 1500 paramedics would be required to participate (Benger et al., 2016). Participation was voluntary, and paramedics were required to attend a 2-hour training session, typically in their own time. Initial recruitment was slow in some regions. It was initially planned that all training would be led by experts in airway management, such as consultants in anaesthesia, critical care or emergency medicine. However, in some areas, demand for larger than expected numbers of (small) training sessions made this logistically difficult. Although there was significant engagement from many medical consultants and senior clinicians, which greatly benefited participants, the aim of consistently including airway experts did not allow sufficient flexibility to recruit paramedics at the required rate.

Paramedics were the units of cluster randomisation, rather than groups or localities as is more often the case in EMS trials (Robinson et al., 2019). This approach avoided the challenges of on-scene patient randomisation, but meant that participating paramedics had to maintain their allocated airway management protocol for the duration of the trial. This method meant that skill fade was potentially a problem, particularly for paramedics allocated to the i-gel arm. In addition, concern was expressed about the potential loss of intubation as a core skill for paramedics and the evidence supporting such a move.

#### Screening of eligible patients

To reduce the risk of bias that could arise as a result of paramedics not being blinded to their airway allocation, it was important that all patients who met the inclusion criteria for the trial (Table 1) were included (Taylor et al., 2016). This necessitated daily screening of the computer-aided dispatch (CAD) system records, which in some areas was up to 70–100 cases per day for the 2-year recruitment period of the trial. In addition, reviews of OHCA audit data were also undertaken to identify eligible patients. Where possible, incidents were cross-referenced with ambulance patient clinical records (PCR). If an eligible but not consciously enrolled patient was identified, the paramedic was consulted to verify eligibility, determine the reason for failure to report the patient and ensure that a trial case report form (CRF) was completed. While almost 70% of required CRFs were returned, 30% were not, requiring an RP to either complete the CRF with the paramedic over the phone or, in the event that the paramedic could not be contacted, to complete it using routine data. Paramedics reported a variety of explanations regarding unreported patients, including misunderstanding or poor recollection of eligibility criteria, forgetting to report and operational pressures (including shift overruns).

**Table 1.** Patient participant inclusion and exclusion criteria for AIRWAYS-2.

Inclusion (all must apply)	Exclusion (if any one applies)
Patient known or believed to be 18 years of age or older	Patient detained by Her Majesty’s Prison Service
Patient has had a non-traumatic cardiac arrest outside hospital	Patient previously recruited to the trial (determined retrospectively)
Patient must be attended by a paramedic who is participating in the trial and is either the first or second paramedic to arrive at the patient’s side	Advanced airway management already in place when AIRWAYS-2 paramedic arrives at patient’s side, inserted by another registered paramedic, a nurse or a doctor (when the first paramedic to arrive is not participating in AIRWAYS-2)
Resuscitation is commenced or continued by ambulance staff or responder	Known to already be enrolled in another pre-hospital randomised trial
	Resuscitation considered inappropriate
	Mouth opening < 2 cm

During the early stages of AIRWAYS-2, a mounting backlog in screening and other activity developed alongside continuing pressure to train more paramedics. Determining which unreported patients were eligible for the trial and pursuing paramedics to complete and return CRFs were the biggest challenges of the out-of-hospital implementation of AIRWAYS-2. The ease with which unreported but possibly eligible patients could be identified varied. While electronic PCRs were being rolled out within some Trusts, they represented only 25% of records accessed for the trial. Even in areas where electronic PCRs were available, relevant data fields were often omitted in favour of free-text entries, limiting the accuracy of automated searching. However, overall, electronic PCRs were easier to identify and interrogate than scanned paper PCRs, the limitations of which have been documented previously (Turner et al., 2008).

Screening processes were further complicated by the inaccurate filing and indexing of some PCRs (paper and electronic), requiring substantial efforts to locate them by cross-referencing multiple information sources. These, along with other factors such as delays in receiving CRFs and the requirement to identify and document a minimal dataset for all 73,000 cardiac arrests which were identified across the four participating regions during the trial period, contributed to the scale of the screening task.

#### Investigation of protocol deviations / reporting errors

In AIRWAYS-2, there were 870 (9.4%) cases where a protocol deviation occurred, either because enrolling paramedics did not perform their allocated intervention first, or because eligible patients were enrolled and/or treated by the wrong paramedic (this was, however, a 10% reduction compared with REVIVE-Airways; Benger et al., 2016). The investigation of apparent protocol deviations required considerable efforts to seek clarity and additional information from both paramedics and patient records (hampered by difficulties in communicating with paramedics). Protocol deviations occurred more frequently in the group of patients who were identified during screening than in those reported by paramedics.

Many paramedics attended few eligible OHCAs in the course of the trial (the mean OHCA attendance per year was three). In addition, trial training stressed that paramedics should have the freedom to deviate from their protocol if they felt it was in the patient’s best interests. One commonly reported obstacle to protocol compliance was perceived clinical and/or hierarchical seniority. On numerous occasions, paramedics reported that no challenge was made to non-trial clinicians who were either already managing a patient’s airway or who wanted to assume control of the airway. In addition, paramedics occasionally attended patients without all necessary equipment, particularly in one Trust where the i-gel was not the routinely used supraglottic airway device. This appears to have occurred most frequently in situations where paramedics were solo responders and/or where dispatch information did not suggest a cardiac arrest. Another common cause of protocol deviation was the presence of significant quantities of vomit or other fluid in a patient’s airway, which typically led to early abandonment of the allocated primary airway management method for paramedics in both trial arms.

### Solutions

The key theme that persisted in tackling the challenges arising during the study was the receptiveness of the chief investigator to issues and suggested solutions, raised by RPs. This empowered the RPs to work together on determining a collective response to problems, as well as allowing each RP the flexibility to adopt the method that worked best within their own Trust. In addition, there were two multidisciplinary investigators’ meetings held during the course of the study which, while logistically challenging to organise, meant that members of the central trial team and participants from each trial site (including principal investigators, research managers, clinical directors, research paramedics and representatives from finance departments) were able to meet face to face.

#### Recruiting and training

The challenge of recruiting paramedics to take part centred on conveying the scientific and clinical rationale for the trial. This is summarised by International Liaison Committee on Resuscitation guidelines: ‘… the evidence to support the use of advanced airway interventions during ALS [advanced life support] remains limited’ (Soar et al., 2015). It was essential to sensitively and effectively engage with the paramedic community. A communication strategy was developed which included posters at ambulance stations, promotional merchandise, newsletters, internal communications, emergency department and station visits by RPs, social media, a trial website, promotion at conferences and events, engagement with opinion leaders, FAQs, training videos and podcasts. Newsletters were embedded in e-mail text because attachments were frequently blocked by firewalls.

Publicity initiatives generally resulted in small but noticeable boosts to paramedic recruitment (particularly e-mails and face-to-face contact at hospital Emergency Departments). However, recruitment remained a challenge, and it was necessary to continue for half of the 2-year patient enrolment period in order to achieve 1523 paramedic participants (it had been anticipated that most paramedic recruitment would be completed prior to the start of patient enrolment).

The challenge of matching those paramedics who expressed an interest in AIRWAYS-2 with training sessions was principally resolved by altering the training model. In one region, sessions were embedded within other service events. However, scope to exploit this strategy was reliant on goodwill from training and operations directorates. For sessions that required paramedics to attend in their own time, the provision of overtime payments, travel costs and attendance certificates helped to mitigate the inconvenience to participants.

To overcome the issues with airway expert availability, it was agreed by the trial management team that having benefited from the experience of training alongside airway experts, the research paramedics were capable of delivering sessions unsupported. This policy facilitated the provision of large numbers of additional small sessions, contributing substantially to the 468 widespread events that were ultimately delivered. However, expert support continued to be sought for larger events where possible.

To assist with skill fade, mid- and end-point training sessions were provided. In addition, there was an online provision for the mid-point training. As with the initial training, overtime payments, travel expenses and attendance certificates were provided. However, these sessions were generally not well attended, with around 10–12% of paramedic participants attending one or more of the training sessions.

#### Screening of eligible patients

The challenges of paramedic recruitment and patient screening were reported back to the trial management group. Crucially, these concerns were listened to and acted upon. Additional support was funded through allocations to individual sites from within the trial budget and in some cases from local Clinical Research Networks (CRNs). However, the quantity and nature of this support varied between sites. The most typical model was to utilise seconded operational staff and those on light duties, but availability of such staff varied. In one Trust, the local CRN was able to assist and provide a research nurse for several months.

#### Investigation of protocol deviations / reporting errors

Close attention was paid to protocol deviations, and there were regular reviews undertaken by the RPs and the trial management team. Part way through the trial, the option of an electronic CRF was introduced. This significantly increased the speed with which forms were received by RPs, enabling rapid query-raising while events were still fresh in the minds of paramedics. Repeated efforts were made to encourage protocol adherence, including laminated aide memoires, a mobile phone web application, refresher training, station champions, posters and e-mails to highlight the protocol and common causes of protocol deviation. However, there were a small number of cases where paramedics had to be withdrawn from the study due to recurrent protocol deviations.

## Discussion

Difficulties in recruiting and training the required number of paramedics in some trial regions appear to have occurred for two particular reasons: concerns of principle, which made paramedics unwilling to be involved (because they considered themselves not to be in equipoise regarding the research question), and difficulty in providing enough convenient training sessions to persuade paramedics to attend them outside their usual working hours. Other potential barriers to paramedic recruitment reported in the literature include extra workload, responsibility and time taken completing paperwork, uncertainty that research is part of a paramedic’s role and concerns that involvement would not be supported by employers (Hargreaves et al., 2014). However, it was not evident that these concerns were major obstacles to participation in AIRWAYS-2.

Despite the emphasis placed on explaining the rationale for the trial, the importance of which was also noted during the PARAMEDIC trial (Pocock et al., 2016), and the fact that the devices used were employed in standard care throughout several of the participating ambulance Trusts, the trial design remained contentious for a number of paramedics. The two chief concerns identified were the perceived impact of the trial on the future availability of tracheal intubation to paramedics, and the perceived adverse effect on skill retention due to the trial protocol. These concerns have been echoed elsewhere as perceived threats to autonomy and professional identity (Hargreaves et al., 2014). One possible way of mitigating the latter would be a periodic intervention crossover trial design (Wang et al., 2016).

Despite successfully undertaking a feasibility trial prior to AIRWAYS-2, the scale of the screening task was under-estimated. Fortunately, a contingency budget did assist with the resolution of this issue, but had this not been available the level of data completeness would have been at risk. Future studies should not underestimate the amount of research paramedic time involved in collating data, even ‘routine’ data, for such studies.

In most clinical trials, research duties such as enrolling patients are conducted by clinicians who have research-specific training and experience. Many of the volunteer paramedics who participated in AIRWAYS-2 had little or no previous exposure to clinical trials. They were nevertheless required to conduct a number of research-related activities without compromising patient care (Lerner et al., 2016), in the context of an OHCA. It is perhaps unsurprising that there were difficulties in recognising eligibility, conducting interventions and reporting patients during and after such events, particularly for paramedics who are infrequently exposed to OHCA.

Evidence from previous out-of-hospital trials suggests that increasing complexity often corresponds with decreasing protocol compliance (Venkataraman et al., 2014). Although efforts were made to keep the processes and procedures required for AIRWAYS-2 uncomplicated, and to challenge recurrent issues, 9.4% of enrolments were subject to protocol deviation. Further simplification of the eligibility criteria (consistent with feedback from the PARAMEDIC trial; Pocock et al., 2016) could encourage even higher levels of protocol adherence, but would require balancing this against consequent changes to perceived acceptability and patient eligibility.

## Conclusion

The main challenges highlighted by the research paramedics employed to facilitate the out-of-hospital phase of the AIRWAYS-2 trial were paramedic recruitment and training, patient screening and the identification and management of protocol deviations. Solutions to these challenges included flexibility and publicity in paramedic recruitment and training, increased out-of-hospital resources for patient screening and improved publicity and education focused on protocol deviations. Large-scale pragmatic cluster randomised trials are being successfully undertaken in out-of-hospital care. However, they require intensive engagement between EMS clinicians and local research paramedics, particularly when the intervention is contentious. Feasibility studies are an important part of research, but they may fail to identify all potential challenges, and flexibility is therefore required to manage unforeseen difficulties.

## Acknowledgements

The AIRWAYS-2 trial team acknowledges the vital contribution of all the paramedics in the East of England, East Midlands, South Western and Yorkshire Ambulance Services who participated in the trial, and all the research staff, at 95 hospitals in England, who collected follow-up data for patients enrolled in the study. We also acknowledge the contribution of the staff of the Clinical Trials and Evaluation Unit, Bristol. This work has not been presented at a conference or published as a conference abstract.

## Author contributions

All authors contributed to the design/delivery of the AIRWAYS-2 trial. All authors drafted or revised this manuscript and approved the final version. JG acts as the guarantor for this article.

## Conflict of interest

RP is editor-in-chief for the *British Paramedic Journal*, but had no editorial control over this submission.

## Ethics

Research Ethics Committee approval was not required for this study, which exclusively involved NHS staff.

## Funding

AIRWAYS-2 was funded by the UK National Institute for Health Research (NIHR) Health Technology Assessment (HTA) programme (project number 12/167/102). The views and opinions expressed herein are those of the authors and do not necessarily reflect those of the HTA, the NIHR, the National Health Service (NHS) or the Department of Health (DoH).

## Trial registration

ISRCTN 08256118. Registered 28 July 2014.
